# Neutrophil to high-density lipoprotein cholesterol ratio predicts adverse cardiovascular outcomes in subjects with pre-diabetes: a large cohort study from China

**DOI:** 10.1186/s12944-022-01695-x

**Published:** 2022-09-03

**Authors:** Shuo-Lin Liu, Bao-Yu Feng, Qi-Rui Song, Ying-Mei Zhang, Shuo-Ling Wu, Jun Cai

**Affiliations:** 1 Department of Cardiology, Zhongshan Hospital, Fudan University, Shanghai Institute of Cardiovascular Diseases, National Clinical Research Center for Interventional Medicine. Key Laboratory of Viral Heart Diseases, National Health Commission. Key Laboratory of Viral Heart Diseases, Chinese Academy of Medical Sciences, Shanghai, 200032 China; 2 Department of Epidemiology and Biostatistics, Institute of Basic Medical Sciences Chinese Academy of Medical Sciences, School of Basic Medicine Peking, Union Medical College, Beijing, 100730 China; 3 Hypertension Center, Fuwai Hospital, State Key Laboratory of Cardiovascular Disease of China, National Center for Cardiovascular Diseases of China, Chinese Academy of Medical Sciences and Peking Union Medical College, Beijing, 100037 China; 4 Department of Cardiology, Kailuan General Hospital, Tangshan, China

**Keywords:** Neutrophil to HDL ratio, Fasting blood glucose, Pre-diabetes, Prognosis

## Abstract

**Background:**

This study aimed to examine whether the neutrophil to high-density lipoprotein cholesterol ratio (NHR) can predict cardiovascular outcomes in normoglycemic individuals with elevated fasting glucose levels.

**Methods:**

A total of 130,801 participants with normal blood glucose levels were enrolled in the Kailuan study. Participants were categorized according to NHR quartiles and further divided into normal glucose regulation (NGR) and pre-diabetes (pre-DM) subgroups. The follow-up endpoint was major adverse cardiovascular events (CVE), including stroke and myocardial infarction.

**Results:**

Over a median of 12.53 (8.95–13.08) years of follow-up, subjects with NHR levels in the highest quartile experienced more CVE than those with NHR levels in the lowest quartile. Multivariate Cox analyses showed that continuous changes in NHR (hazard ratio, 1.21; 95% confidence interval [CI], 1.15–1.28) and the highest quartile of NHR (hazard ratio, 1.30; 95% CI, 1.21–1.39) were independent predictors of CVE (all *P* < 0.001). Furthermore, when participants were categorized by both NHR quartile and glucose metabolism status, the NHR level in the highest quartile plus pre-DM group was associated with a 1.60-fold (95% CI, 1.38–1.86; *P* < 0.001] higher risk of CVE than that in the lowest quartile plus normoglycemic group. Significantly, the addition of NHR only, presence of pre-DM only, or combination of NHR and pre-DM to the prediction algorithm, including traditional risk factors, improved the C-statistic by 0.19, 0.05, and 0.23 (all *P* < 0.001).

**Conclusions:**

Elevated NHR or fasting blood glucose level were independently associated with a higher risk of CVE among normoglycemic individuals. Moreover, pre-DM participants with high NHR levels tended to have worse prognosis, suggesting that NHR could provide greater risk stratification value than traditional risk factors for subjects with pre-DM.

**Supplementary Information:**

The online version contains supplementary material available at 10.1186/s12944-022-01695-x.

## Background

Cardiovascular diseases (CVD), including myocardial infarction and stroke, are the leading cause of death worldwide [1, 2]. The primary cause of vascular disease is the presence of atherosclerotic lesions in the arterial wall. Atherosclerosis is a dynamic development characterized by plaque formation and destabilization driven by multiple pathological factors [3]. Inflammation, lipid abnormalities, and glucose metabolism dysfunction play pivotal roles in this complex process [1].

Neutrophils potentiate cardiac injury through multiple mechanisms, including proinflammatory cell recruitment and cytokine release [4]. Elevated circulating neutrophils are significantly associated with greater arterial wall stiffness [5] and an increased risk of adverse cardiovascular outcomes, including stroke, rapidly progressing heart failure, and acute coronary syndrome [4–6]. Furthermore, high-density lipoprotein cholesterol (HDL-C) particles inhibit endothelial cells in response to inflammation and oxidative stress by suppressing neutrophil activation and low-density cholesterol (LDL-C) oxidation [7–10]. Moreover, activated neutrophils curb the crucial role of HDL-C in reverse cholesterol transport [11]. The neutrophil to HDL-C ratio (NHR) is an inflammatory and lipid metabolic marker that predicts adverse cardiovascular outcomes. A few observational studies have reported that NHR assisted in the risk profiling of adverse cardiovascular events (CVE) such as long-term mortality, recurrent myocardial infarction (MI), intravenous thrombolysis, and severe artery stenosis in ischemic stroke or MI populations [12–14]. However, the risk prediction value of NHR in low-risk populations has not yet been investigated.

Pre-diabetes mellitus (pre-DM), a glucose metabolic state over normal glucose regulation (NGR) but below the threshold of diabetes, includes impaired fasting glycemia and/or impaired glucose tolerance [15]. While increased fasting blood glucose (FBG) has long been recognized as a predictor of adverse CVE [16–18], accumulating evidence has revealed a significant association between elevated FBG levels in the normoglycemic range with subclinical inflammation [19], arterial stiffness [20], and a high incidence of CVD [21, 22]. Therefore, a healthy general population with pre-DM warrants further risk stratification for a better prognosis [23]. Moreover, glucose metabolism disorders and insulin resistance significantly accelerate the inflammatory response, endothelial dysfunction, and atherosclerosis progression [24, 25]. Thus, the NHR may identify a higher cardiovascular risk among individuals with pre-DM.

Thus, this follow-up study of a community-based Chinese population cohort sought to explore the prognostic value of NHR on cardiovascular outcomes in normoglycemic individuals with or without pre-DM.

## Materials and methods

### Study design and population

**Fig. 1:**
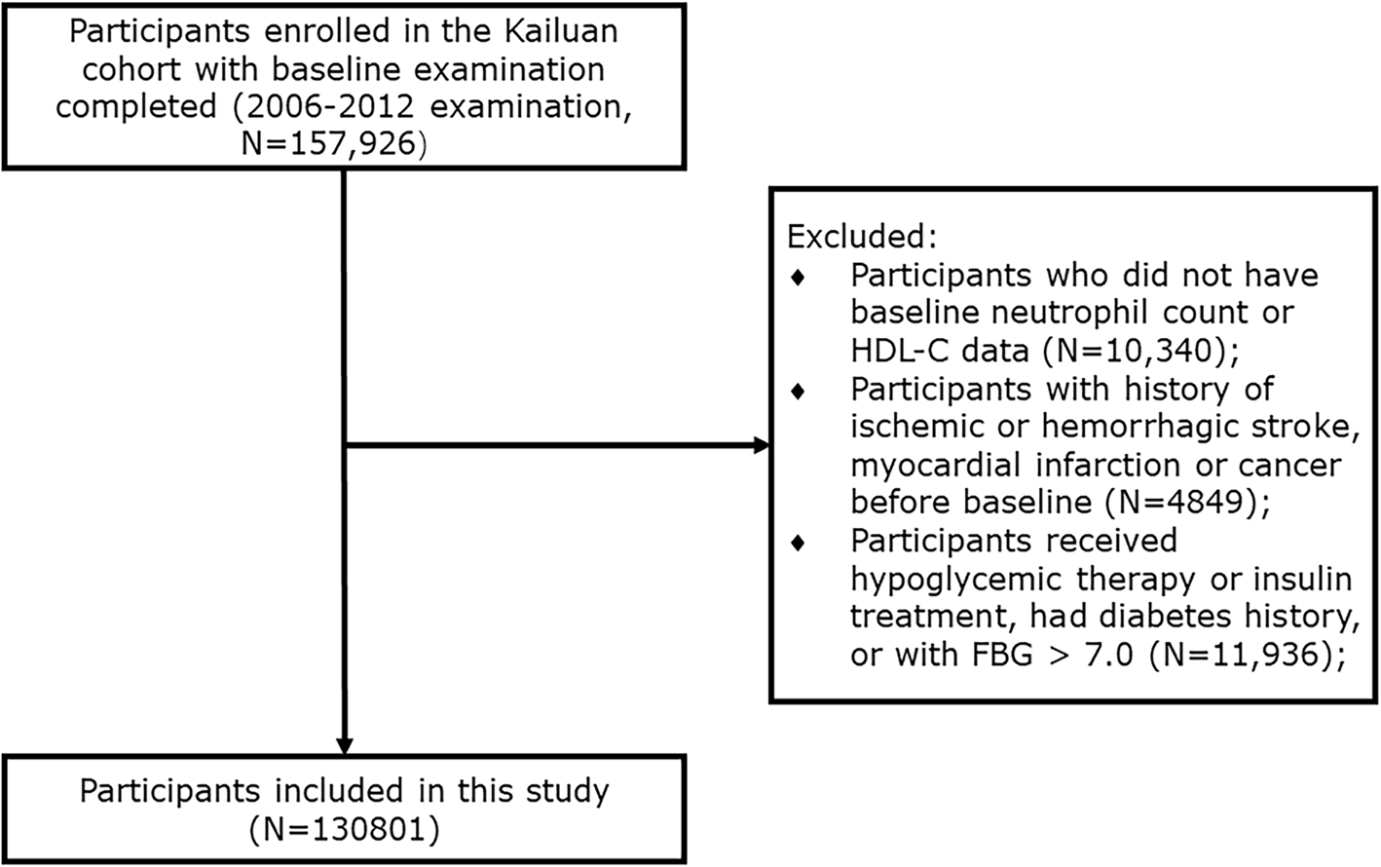
Flowchart of the study population

This Kailuan study (trial registration number, ChiCTR-TNC-1100148; trial registration site, http://www.chictr.org.cn/index.aspx) is a prospective cohort investigation conducted in communities in Tangshan City, Hebei Province, China [26]. The study was initiated from January 2006 to December 2012, and follow-up examinations were performed biennially, in which all participants underwent questionnaires, laboratory tests, and physical examinations. Meanwhile, cardiovascular, cerebrovascular, and related non-communicable diseases were monitored through provincial vital statistics data, the Tangshan medical insurance system, and the Kailuan Group Medical Insurance Information System. Overall, 157,926 individuals aged > 18 years participated in the Kailuan study. Of all recruited individuals, after the exclusion of 10,340 subjects for whom no neutrophil or HDL-C level information was available and 4,849 who had a medical history of malignant tumor, myocardial infarction (MI), or stroke, 142,737 individuals were enrolled. Furthermore, 11,936 individuals who received hypoglycemic therapy or insulin treatment or those with an FBG ≥ 7.0 mmol/L were excluded. Thus, a total of 130,801 individuals were included in the final analysis (Fig. 1).

This study was approved by the ethics committee of Kailuan Hospital and conformed to the Declaration of Helsinki. All participants signed an informed consent form.

### Definition of diabetes and pre-diabetes

Blood samples from all participants were acquired within 24 h of fasting after hospital admission. According to American Diabetes Association criteria [27], pre-DM was defined as an FBG level of 5.60–6.99 mmol/L, while a normal NGR was defined as an FBG level of <5.60.

### Outcomes

CVE was defined previously [28] as a composite of stroke and MI until December 31, 2019 [29]. The endpoints of this study were the first appearance of CVE, which was identified by medical staff from the provincial vital statistics data, Tangshan medical insurance system, and Kailuan Group Medical Insurance Information System during the semiannual interviews. An experienced panel of cardiologists gathered and examined the medical records. CVE were determined according to the International Classification of Diseases, Tenth Revision. MI was ascertained based on characteristic clinical symptoms, elevated myocardial enzyme activity, and changes in serial electrocardiography (I12) [30]. Stroke was defined based on characteristic clinical symptoms and signs, and brain imaging examinations (I61, I63) [31]. All-cause mortality was determined by cardiologists from vital statistical offices.

### Laboratory tests

The concentrations of the lipid parameters were determined using an automatic biochemistry analyzer (Hitachi 747, Tokyo, Japan) [28]. White blood cell counts were measured using an XE-1200 automated hematology analyzer (Sysmex, Kobe, Japan). Plasma FBG levels were determined using a highly sensitive and specific commercial sandwich enzyme immunoassay (BI-20082H; Biomedica, Wien, Austria). Hyperlipidemia was determined by medical history review. Physical activity was defined as moderate-intensity exercise ≥3 times/week for ≥30 min [28].

### Statistical analysis

The statistical analysis was conducted using SPSS version 22.0 and R language version 3.5.2. Multiple imputation methods were used to compensate for missing data [32]. The values are expressed as mean±SD, median (25^th^ to 75^th^ percentiles) for continuous variables and number (percentage) for categorical variables. The Kolmogorov–Smirnov test was performed to determine the distribution of the parameters. Intergroup comparisons of the continuous and categorical variables were examined using Student’s t-test and the chi-squared test, respectively. Cox proportional hazards models were used to examine the association between NHR or FBG level and the occurrence of CVE with the adjustment for traditional risk factors of CVD, including age, sex, smoking, drinking, education, physical status, obesity, hypertension, dyslipidemia, and C-reactive protein (CRP). In addition, a sensitivity analysis of the association of NHR or FBG with the prediction of CVE (with separate adjustment for each of the other variables in the multivariate model) was performed. The improved predictive efficiency of NHR and FBG over the clinical risk factors was evaluated using C-statistics and △ C-statistics. Kaplan-Meier curves for CVE were estimated, and differences among groups were compared using the log-rank test. Statistical significance was set at *P* < 0.05.

## Results

### Baseline characteristics

This study included 130,801 eligible participants. The baseline clinical data according to NHR quartile are presented in Table 1. The average NHR in Q1, Q2, Q3, and Q4 was 1.5±0.3, 2.2±0.2, 2.9±0.2, and 4.5±1.3, respectively (*P* <0.001). Participants in higher NHR quartiles were significantly younger, were more likely male and smokers, were inactive, had higher blood pressures, higher body mass index, FBG, triglyceride, LDL-C, CRP concentrations, and neutrophil counts and had lower HDL-C concentrations than those in the lowest NHR quartile (all *P* < 0.001). Furthermore, the percentages of individuals with higher education levels and those with hypertension or dyslipidemia also increased in the higher quartiles of the NHR group (all *P* < 0.001).

**Table 1:** Clinical characteristics of participants according to NHR levels

Variables	Total (*N*=130801)	Q1 (*N*=33676)	Q2 (*N*=32922)	Q3 (*N*=32436)	Q4 (*N*=31767)	*P* Value
Age (years)	48.3±13.9	49.4±13.6	48.6±13.8	48.0±14.1	46.9±14.2	<0.001
Male, n (%)	104536 (79.9)	24204 (71.9)	26087 (79.2)	26718 (82.4)	27527 (86.7)	<0.001
Physical activity, n (%)						<0.001
Inactive	19039 (14.6)	4590 (13.6)	4638 (14.1)	4694 (14.5)	5117 (16.1)	
Moderate active	93386 (71.4)	24275 (72.1)	23857 (72.5)	23184 (71.5)	22070 (69.5)	
Active	18376 (14.0)	4811 (14.3)	4427 (13.4)	4558 (14.1)	4580 (14.4)	
Above high school education level, n (%)	34818 (26.6)	8712 (25.9)	8483 (25.8)	8675 (26.7)	8948 (28.2)	<0.001
Current or previous smoking, n (%)	51802 (39.6)	11459 (34.0)	12323 (37.4)	13302 (41.0)	14718 (46.3)	<0.001
Current or previous drinking, n (%)	22194(17.0)	5753(17.1)	5512(16.7)	5482(16.9)	5447(17.1)	0.507
BMI (kg/m^2^)	24.8±3.5	24.1±3.4	24.7±3.4	25.1±3.5	25.5±3.6	<0.001
SBP (mmhg)	128.1±20.0	126.9±20.2	128.2±20.3	128.6±20.0	128.6±19.6	<0.001
DBP (mmhg)	82.8±11.5	81.9±11.5	82.8±11.5	83.1±11.4	83.5±11.5	<0.001
FBG (mmol/L)	5.1±0.7	5.1±0.7	5.1±0.7	5.1±0.7	5.1±0.7	<0.001
TG (mmol/L)	1.2 (0.9-1.8)	1.1 (0.8-1.6)	1.2 (0.9-1.8)	1.3 (0.9-1.9)	1.4 (1.0-2.1)	<0.001
TC (mmol/L)	4.9±1.1	5.0±1.1	4.9±1.1	4.8±1.1	4.7±1.0	<0.001
HDL-C (mmol/L)	1.5±0.4	1.8±0.3	1.5±0.3	1.4±0.3	1.2±0.3	<0.001
LDL-C (mmol/L)	2.4±0.9	2.4±0.9	2.4±0.9	2.5±0.9	2.5±0.9	<0.001
CRP, mg/L	0.9 (0.3-2.2)	0.7 (0.3-1.8)	0.8 (0.3-2.0)	0.9 (0.4-2.3)	1.2 (0.5-2.9)	<0.001
Hypertension, n (%)	49987 (38.2)	12173 (36.1)	12819 (38.9)	12758 (39.3)	12237 (38.5)	<0.001
Dyslipidemia, n (%)	41155 (31.5)	7823 (23.2)	8566 (26.0)	9846 (30.4)	14920 (47.0)	<0.001
Neutrophils, 10^9/L	3.8±1.3	2.6±0.6	3.4±0.7	4.1±0.8	5.2±1.4	<0.001
NHR	2.8±1.3	1.5±0.3	2.2±0.2	2.9±0.2	4.5±1.3	<0.001

*TG* Triglyceride, *TC* Total cholesterol, *HDL-C* High-density lipoprotein cholesterol, *LDL-C* Low-density lipoprotein cholesterol, *BMI* Body mass index, *CRP* C-reactive protein, *SBP* Systolic blood pressure, *DBP* Diastolic blood pressure, *NHR* Neutrophil to HDL-C level ratio

### Association between NHR level and cardiovascular outcomes

Over a median of 12.53 (8.95–13.08) years of follow-up, 7,013 CVE occurred (1,552 MI, 5,602 strokes). The corresponding prevalence of CVE in Q2, Q3, and Q4 of the NHR level were 5.33%, 5.81%, and 5.86%, respectively (*P* < 0.001). According to univariate Cox proportional hazard regression analysis, per SD change in NHR (hazard ratio [HR], 1.12; 95% confidence interval [CI], 1.09-1.14; *P* < 0.001) and continuous change in NHR (HR, 1.29; 95% CI, 1.22–1.36; *P* < 0.001) were independently associated with increased CVE. Furthermore, Q2 of NHR (HR, 1.21; 95% CI, 1.13–1.29; *P* < 0.001), Q3 of NHR (HR, 1.35; 95% CI, 1.27–1.45; *P* < 0.001), and Q4 of NHR (HR, 1.41; 95% CI, 1.32–1.51; *P* < 0.001) were significantly associated with an increased risk of CVE compared with Q1 of NHR. The adjustment for potential confounding factors, including age, male sex, smoking, drinking, education, physical activity, obesity, hypertension, dyslipidemia, and CRP levels in models 1 and 2, did not change this relationship (Table 2). Moreover, NHR was also associated with all-cause mortality in uni- and multivariate Cox proportional hazard regression analyses (Supplementary Table 1).

**Table 2:** Relation of the NHR level and cardiovascular outcomes in univariate and multivariate survival analysis

		HR (95% CI)					
Variables	Events/subjects	Crude model	*P* value	model 1	*P* value	model 2	*P* value
NHR (per SD change)^a^	7013/130801	1.12(1.09-1.14)	<0.001	1.13(1.11-1.16)	<0.001	1.09(1.06-1.11)	<0.001
NHR (continuous change)^a^	7013/130801	1.29(1.22-1.36)	<0.001	1.32(1.25-1.39)	<0.001	1.21(1.15-1.28)	<0.001
NHR							
Q1	1512/33676	Reference		Reference		Reference	
Q2	1755/32922	1.21(1.13-1.29)	<0.001	1.20(1.12-1.29)	<0.001	1.14(1.07-1.23)	<0.001
Q3	1884/32436	1.35(1.27-1.45)	<0.001	1.36(1.27-1.46)	<0.001	1.26(1.18-1.35)	<0.001
Q4	1862/31767	1.41(1.32-1.51)	<0.001	1.45(1.36-1.55)	<0.001	1.30(1.21-1.39)	<0.001

^a^Log-transformed NHR; Model 1 adjusted for age, gender, smoker, drinker, education, physical activity. Model 2 adjusted for age, gender, smoker, drinker, education, physical activity, obesity, hypertension, dyslipidemia, C-reactive protein and fasting blood glucose. *NHR* Neutrophil to high-density lipoprotein cholesterol level ratio

**Fig. 2:**
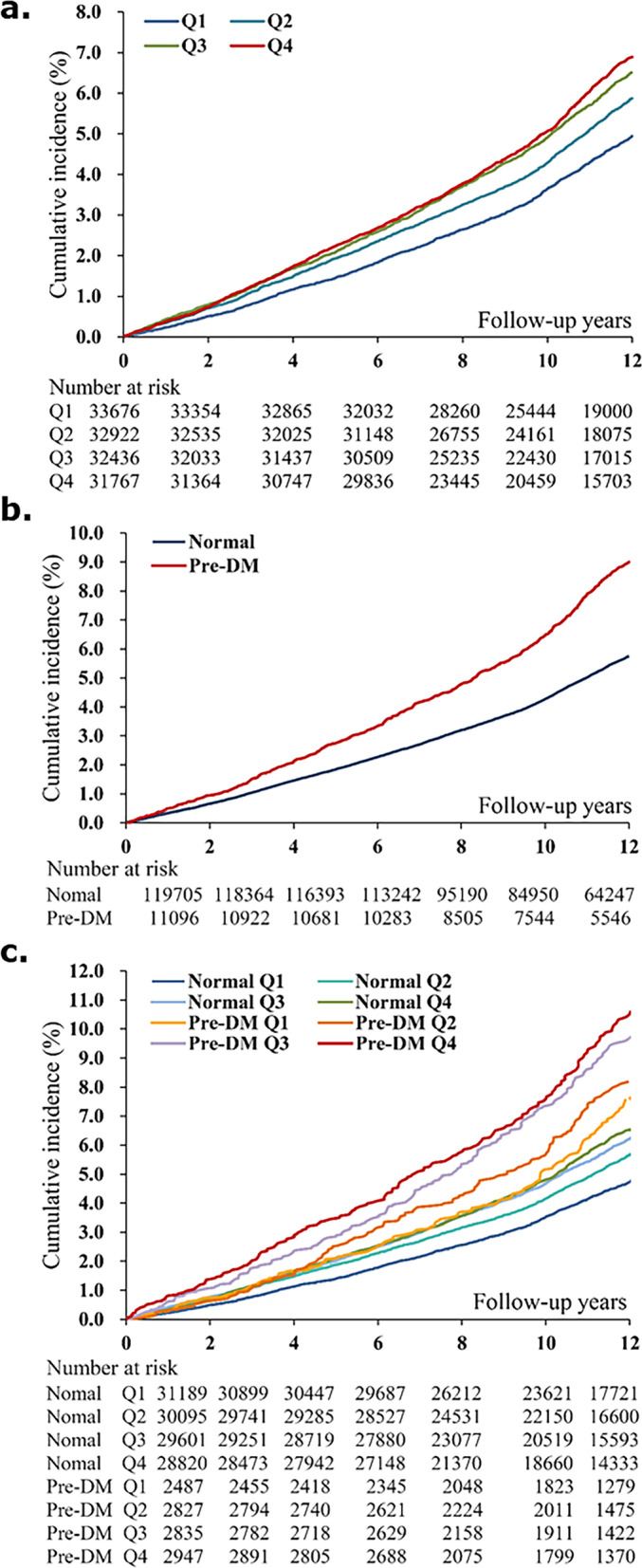
Kaplan–Meier curves by neutrophil to high-density lipoprotein cholesterol ratio (NHR) (**a**); glucose metabolism status (**b**); and NHR combined with glucose metabolism status (**c**)

Kaplan-Meier analysis with the log-rank test demonstrated that patients in higher NHR quartiles had a significantly higher risk of CVE. Individuals in the NHR Q4 had the highest cumulative incidence of CVE (*P <* 0.001; Fig. 2a).

### Pre-diabetes, NHR, and cardiovascular outcomes

During the follow-up period, the pre-DM group had a higher incidence of CVE (7.84%) than the NGR group (5.13%). The univariate Cox regression model showed that a per SD change in FBG and continuous change in FBG were associated with a 1.13-fold (95% CI, 1.10–1.16; *P* < 0.001) and 1.20-fold (95% CI, 1.16–1.24; *P* < 0.001) higher risk of CVE, respectively. Furthermore, patients with pre-DM were at increased risk of CVE (HR, 1.58; 95% CI, 1.47–1.69; *P* < 0.001). The data indicated that the association between MHR and CVE was not significantly altered after the adjustment for other confounders (Table 3). Pre-DM was also associated with a higher risk of all-cause mortality in the uni- and multivariate Cox regression models (Supplementary Table 2).

**Table 3:** Increased FBG is associated with cardiovascular outcomes in normoglycemic individuals

		HR (95% CI)					
Variables	Events/subjects	Crude model	*P* value	model 1	*P* value	model 2	*P* value
FBG (per SD change)^a^	7013/130801	1.13(1.10-1.16)	<0.001	1.09(1.07-1.11)	<0.001	1.04(1.02-1.06)	0.001
FBG (continuous change)^a^	7013/130801	1.20(1.16-1.24)	<0.001	1.14(1.10-1.18)	<0.001	1.06(1.02-1.09)	0.001
NGR	6143/119705	Reference		Reference		Reference	
Pre-DM	870/11096	1.58(1.47-1.69)	<0.001	1.35(1.26-1.45)	<0.001	1.19(1.11-1.28)	<0.001

^a^Log-transformed NHR; Model 1 adjusted for age, gender, smoker, drinker, education, physical activity. Model 2 adjusted for age, gender, smoker, drinker, education, physical activity, obesity, hypertension, dyslipidemia, C-reactive protein and fasting blood glucose. *FBG* Fasting blood glucose, *NGR* Normal glucose regulation, *Pre-DM* Pre-diabetes mellitus

CVE risk was assessed according to both NHR quartile and the presence of pre-DM. Univariate Cox regression analysis showed that NHR was positively and significantly associated with CVE from NGR to pre-DM relative to NHR Q1 of the NHR and NGR groups (*P* for trend < 0.001; Table 4). Additionally, the risk of CVE in pre-DM with Q3 of NHR and pre-DM with Q4 of NHR was up to 2.13-fold (95% CI, 1.85–2.44; *P* < 0.001) and 2.30-fold (95% CI, 2.02–2.63; *P* < 0.001) higher, respectively. This association was not altered in multivariate-adjusted models 1 and 2 (Table 4). The same trend of NHR for all-cause mortality in patients with pre-DM was observed (Supplementary Table 3).

**Table 4:** NHR levels in relation to cardiovascular outcomes in patients with pre-diabetes

		HR (95% CI)					
Variables	Events/subjects	Crude model	*P* value	model 1	*P* value	model 2	*P* value
NGR							
NHR (per SD change)^a^	6143/119705	1.11(1.08-1.13)	<0.001	1.12(1.09-1.15)	<0.001	1.08(1.05-1.11)	<0.001
NHR (continuous change)^a^	6143/119705	1.26(1.19-1.33)	<0.001	1.30(1.23-1.37)	<0.001	1.19(1.12-1.26)	<0.001
Pre-DM							
NHR (per SD change)^a^	870/11096	1.18(1.10-1.26)	<0.001	1.18(1.10-1.26)	<0.001	1.16(1.08-1.24)	<0.001
NHR (continues change)^a^	870/11096	1.44(1.24-1.68)	<0.001	1.45(1.24-1.69)	<0.001	1.40(1.19-1.64)	<0.001
NGR							
NHR Q1	1351/31189	Reference		Reference		Reference	
NHR Q2	1551/30095	1.21 (1.12-1.30)	<0.001	1.21(1.13-1.30)	<0.001	1.15(1.07-1.24)	<0.001
NHR Q3	1640/29601	1.34 (1.25-1.44)	<0.001	1.36(1.26-1.46)	<0.001	1.26(1.17-1.35)	<0.001
NHR Q4	1601/28820	1.38 (1.29-1.49)	<0.001	1.43(1.33-1.54)	<0.001	1.28(1.19-1.38)	<0.001
Pre-DM							
NHR Q1	161/2487	1.53 (1.30-1.81)	<0.001	1.36(1.16-1.61)	<0.001	1.17(0.98-1.39)	0.083
NHR Q2	204/2827	1.74 (1.50-2.01)	<0.001	1.49(1.29-1.73)	<0.001	1.27(1.08-1.49)	0.004
NHR Q3	244/2835	2.13 (1.85-2.44)	<0.001	1.80(1.57-2.06)	<0.001	1.48(1.27-1.72)	<0.001
NHR Q4	261/2947	2.30 (2.02-2.63)	<0.001	2.01(1.76-2.29)	<0.001	1.60(1.38-1.86)	<0.001
P for trend			<0.001		<0.001		<0.001

^a^Log-transformed NHR; Model 1 adjusted for age, gender, smoker, drinker, education, physical activity. Model 2 adjusted for age, gender, smoker, drinker, education, physical activity, obesity, hypertension, dyslipidemia, C-reactive protein and fasting blood glucose. *NHR* Neutrophil to high-density lipoprotein cholesterol level ratio, *NGR* Normal glucose regulation, *pre-DM* Pre-diabetes mellitus

Kaplan-Meier curves indicated that individuals with pre-DM had lower cumulative event-free survival rates (Fig. 2b). Moreover, the risk of CVE was further stratified according to NHR quartile and the occurrence of pre-DM. Participants in the highest NHR quartile and pre-DM groups had the lowest cumulative incidence of CVE (*P* for trend *<* 0.01; Fig. 2c).

### Incremental predictive value of NHR and pre-DM for cardiovascular outcomes

As shown in Table 5, the crude model of traditional risk factors had a C-statistic of 0.752 (95% CI, 0.747–0.757). The addition of NHR to the original model increased the C-statistic to 0.754 (95% CI, 0.749–0.759; ΔC-statistic, 0.19; *P* < 0.001). Similarly, adding pre-DM to the original model increased the C-statistic to 0.753 (95% CI, 0.748–0.758; ΔC-statistic, 0.05; *P* < 0.001). Furthermore, significant improvement in the model prediction for CVE was observed with the addition of NHR and pre-DM to the original model (C-statistic, 0.754; 95% CI, 0.749–0.759; ΔC-statistic, 0.23; *P* < 0.001).

**Table 5:** Incremental predictive values of NHR and prediabetes for cardiovascular outcomes

Models	C-statistic (95% CI)	ΔC-statistic (%)	*P* value
original model	0.752(0.747-0.757)	-	-
original model +NHR	0.754(0.749-0.759)	0.19	<0.001
original model + pre-DM	0.753(0.748-0.758)	0.05	<0.001
original model +NHR +pre-DM	0.754(0.749-0.759)	0.23	<0.001

*NHR* Neutrophil to high-density lipoprotein cholesterol level ratio, *pre-DM* Pre-diabetes mellitus

## Discussion

### Main findings

This community-based cohort study of 130,801 normoglycemic individuals explored the effect of NHR on cardiovascular outcomes in subjects with or without pre-DM. Cox regression analysis showed that higher NHR or FBG levels were significantly associated with long-term adverse cardiovascular risk. Furthermore, pre-DM individuals with high NHR levels tended to have worse prognosis. Notably, the addition of NHR and pre-DM to the original model significantly improved the risk prediction beyond the original risk factor model. This study demonstrated that NHR, as a user-friendly factor, could assist in the risk stratification of individuals with pre-DM.

### Predictive values of NHR for CVE

Discovering novel user-friendly laboratory indices could help stratify risk and identify therapeutic strategies [33]. Inflammation and dyslipidemia are two interactive pathological processes that drive the initiation and progression of atherosclerotic cardiovascular disease, while neutrophils drive its early inflammatory response [34, 35]. Neutrophil accumulation was identified at the site of atherosclerotic lesions and contributes to plaque instability [36]. The increased release of myeloperoxidase from neutrophils can aggravate atherosclerotic lesions [37, 38]. Dyslipidemia is causally associated with atherosclerosis progression. HDL-C displays an anti-atherogenic effect by transporting excess cholesterol from adipocytes or macrophages back to the liver [39]. Patients with lower HDL-C levels appear to be at higher risk of cardiovascular mortality and CVE in the general population [10, 40].

Additionally, HDL-C possesses the capacity to suppress the inflammatory response and oxidation [10, 41]. HDL-C can blunt neutrophil activation, adhesion, and migration [8]. Therefore, NHR was proposed as a novel index to evaluate the effects of inflammation and dyslipidemia on cardiovascular disease prognosis. Recent studies revealed that NHR is significantly elevated in multiple pathological states and contributes to the development of several diseases [12–14, 42]. NHR is significantly increased in patients with severe stroke and acts as an independent predictor of short-term outcomes [12].

A recent study of 404 patients who underwent coronary angiography demonstrated that NHR was associated with severe coronary stenosis [14]. Another study reported that NHR predicted long-term mortality and recurrent MI in elderly patients with acute MI [13]. Moreover, a cohort study of 983 peritoneal dialysis patients found that NHR had better prognostic value for all-cause mortality and new-onset CVE in patients with high cardiovascular risk [43]. However, whether NHR can predict long-term cardiovascular risk in a low-risk population has not been explored. Importantly, the present study showed that elevated NHR levels were significantly associated with a higher risk of CVE. Thus, NHR has important prognostic value in large community-based populations.

Individuals with pre-DM require precise cardiovascular risk stratification [23]. Thus, the identification of novel circulating biomarkers is crucial for this population. This study explored the predictive performance of NHR for cardiovascular risk in individuals with pre-DM. The present study demonstrated that, among normoglycemic individuals, pre-DM plus elevated NHR levels were associated with a 2.30-fold higher risk of adverse cardiovascular outcomes than NGR and low NHR levels. Moreover, combining NHR with pre-DM offers incremental predictive value over traditional risk factors in the general population, suggesting that subclinical inflammation influences the cardiovascular prognosis of patients with impaired fasting glucose.

The concrete mechanism of this phenomenon is as follows. Alterations in vascular homeostasis caused by endothelial dysfunction are the most apparent characteristics of diabetic angiopathy that facilitate an inflammatory response and finally lead to atherosclerosis [25]. Specifically, long-term exposure to impaired blood glucose coupled with dyslipidemia and subclinical inflammation contribute to the pathophysiological progression of hyperglycemia-related macrovascular disorders [44]. Abnormal glycemic concentrations induce oxidative stress and overload free radicals in endothelial cells, triggering multiple molecular mechanisms involving nuclear factor-kappa B (NF-κB)-mediated vascular inflammation and protein kinase C activation, which contributes to vasoconstriction and platelet adhesion [45]. In addition, NF-kB signaling activation upregulates multiple proinflammatory genes [44]. Therefore, NHR is a reliable and incremental prognostic biomarker that can aid the cardiovascular risk stratification in patients with pre-DM.

### Comparisons with other studies and what does the current work add to the existing knowledge

Previous studies demonstrated that NHR is associated with adverse cardiovascular outcomes in high-risk patients with coronary disease or stroke[12–14]. However, whether it is associated with a higher cardiovascular risk in the low-risk general population remains unclear. The current study revealed that NHR could predict CVE in low-risk individuals. Moreover, pre-DM participants with high NHR levels appeared to have a higher cardiovascular risk.

### Strength and study limitations

This study has several strengths. The present study revealed a novel cardiovascular risk prediction role for NHR in a low-risk population. In addition, this cohort study comprised a large population of over 130,000 Chinese adults and was followed up for a median 12.53 years, which confirms its credibility. However, this study also has several limitations. First, its observational and prospective design may have been influenced by potential biases from the non-random assignment of exposures. Second, its analyses were based on the baseline measurements of neutrophils and FBG without oral glucose tolerance testing and glycosylated hemoglobin A1c level examination; therefore, patients with new-onset diabetes during the follow-up period may have been missed. Third, although individuals with an acute infectious status were excluded during the physical examination, the present study had no data about infectious disease.

## Conclusions

In the general population, higher FBG levels, even within the normal range, and elevated NHR are associated with a higher cardiovascular risk. Our findings suggest that NHR provides critical value for risk stratification and prognosis prediction for individuals with pre-DM.

## Supplementary Information

**Additional file 1:** **Supplement Table 1.** Relation of the NHR level and all-cause mortality in univariate and multivariate survival analysis. **Supplement Table 2.** Increased FBG is associated with all-cause mortality in normoglycemic individuals. **Supplement Table 3.** NHR levels in relation to all-cause mortality in patients with pre-diabetes.

## Data Availability

The datasets used and/or analyzed during the current study are available upon reasonable request from the corresponding author.

## Abbreviations

BMI: Body mass index
CABG: Coronary artery bypass grafting
CI: Confidence interval
CRP: C-reactive protein
CVE: Cardiovascular events
DM: Diabetes mellitus
FBG: Fasting blood glucose
HDL-C: High-density lipoprotein cholesterol
HR: Hazard ratio
LDL-C: Low-density lipoprotein cholesterol
MI: Myocardial infarction
NF-κB: Nuclear factor-kappa B
NGR: Normal glucose regulation
NHR: Neutrophil to high-density lipoprotein cholesterol ratio
