# Supplementary material for: Neutrophil to high-density lipoprotein cholesterol ratio predicts adverse cardiovascular outcomes in subjects with pre-diabetes: a large cohort study from China
Source: Lipids Health Dis. 2022 Sep 3;21:86. doi: 10.1186/s12944-022-01695-x (PMC9441053; doi:10.1186/s12944-022-01695-x)
Supplement: Supplementary file 1 — Additional file 1: Supplement Table 1. Relation of the NHR level and all-cause mortality in univariate and multivariate survival analysis. Supplement Table 2. Increased FBG is associated with all-cause mortality in normoglycemic individuals. Supplement Table 3. NHR levels in relation to all-cause mortality in patients with pre-diabetes. [file 12944_2022_1695_MOESM1_ESM.docx]

Supplement Table 1. Relation of the NHR level and all-cause mortality in univariate and multivariate survival analysis.

|  |  | HR (95% CI) | | | | | |
| --- | --- | --- | --- | --- | --- | --- | --- |
| Variables | Events/subjects | Crude model | P value | model 1 | P value | model 2 | P value |
| NHR (per SD change)* | 10271/130801 | 1.04(1.02-1.06) | <0.001 | 1.06(1.04-1.08) | <0.001 | 1.05(1.03-1.07) | <0.001 |
| NHR (continuous change)* | 10271/130801 | 1.08(1.04-1.13) | <0.001 | 1.15(1.10-1.20) | <0.001 | 1.12(1.07-1.17) | <0.001 |
| NHR | / |  |  |  |  |  |  |
| Q1 | 2575/33676 | Reference |  | Reference |  | Reference |  |
| Q2 | 2581/32922 | 1.04(0.98-1.10) | 0.17 | 1.05(0.99-1.10) | 0.107 | 1.03(0.98-1.09) | 0.279 |
| Q3 | 2631/32436 | 1.10(1.05-1.17) | <0.001 | 1.13(1.07-1.19) | <0.001 | 1.10(1.05-1.17) | <0.001 |
| Q4 | 2484/31767 | 1.10(1.04-1.16) | 0.001 | 1.18(1.11-1.25) | <0.001 | 1.14(1.08-1.21) | <0.001 |

Note: TG: triglyceride; TC: total cholesterol; HDL-C, high-density lipoprotein cholesterol; LDL-C: low-density lipoprotein cholesterol; BMI, body mass index; CRP, C-reactive protein; SBP, systolic blood pressure; DBP, diastolic blood pressure; NHR, neutrophil to HDL-C level ratio.

Supplement Table 2. Increased FBG is associated with all-cause mortality in normoglycemic individuals.

|  |  | HR (95% CI) | | | | | |
| --- | --- | --- | --- | --- | --- | --- | --- |
| Variables | Events/subjects | Crude model | P value | model 1 | P value | model 2 | P value |
| FBG (per SD change)* | 10271/130801 | 1.02(1.00-1.04) | 0.027 | 1.01(0.99-1.03) | 0.173 | 1.00(0.98-1.02) | 0.854 |
| FBG (continuous change)* | 10271/130801 | 1.03(1.00-1.06) | 0.027 | 1.02(0.99-1.05) | 0.173 | 1.00(0.98-1.03) | 0.854 |
| FBG level | / |  |  |  |  |  |  |
| NGR | 9164/119705 | Reference |  | Reference |  | Reference |  |
| Pre-DM | 1107/11096 | 1.33(1.25-1.41) | <0.001 | 1.12(1.05-1.19) | <0.001 | 1.08(1.01-1.15) | 0.022 |

Note: ^*^ Log-transformed NHR; Model 1 adjusted for age, gender, smoker, drinker, education, physical activity. Model 2 adjusted for age, gender, smoker, drinker, education, physical activity, obesity, hypertension, dyslipidemia, C-reactive protein and fasting blood glucose. FBG, fasting blood glucose; NGR, normal glucose regulation; Pre-DM, pre-diabetes mellitus.Supplement Table 3. NHR levels in relation to all-cause mortality in patients with pre-diabetes.

|  |  | HR (95% CI) | | | | | |
| --- | --- | --- | --- | --- | --- | --- | --- |
| Variables | Events/subjects | Crude model | P value | model 1 | P value | model 2 | P value |
| NGR |  |  |  |  |  |  |  |
| NHR (per SD change)* | 9164/119705 | 1.03(1.01-1.05) | 0.006 | 1.06(1.04-1.08) | <0.001 | 1.05(1.03-1.07) | <0.001 |
| NHR (continuous change)* | 9164/119705 | 1.07(1.02-1.12) | 0.006 | 1.15(1.09-1.20) | <0.001 | 1.11(1.06-1.17) | <0.001 |
| Pre-DM | / |  |  |  |  |  |  |
| NHR (per SD change) * | 1107/11096 | 1.07(1.01-1.14) | 0.022 | 1.06(1.00-1.13) | 0.061 | 1.07(1.00-1.14) | 0.047 |
| NHR (continues change) * | 1107/11096 | 1.18(1.02-1.35) | 0.022 | 1.15(0.99-1.32) | 0.061 | 1.16(1.00-1.34) | 0.047 |
| NGR | / |  |  |  |  |  |  |
| NHR Q1 | 2351/31189 | Reference |  | Reference |  | Reference |  |
| NHR Q2 | 2291/30095 | 1.02(0.97-1.08) | 0.436 | 1.04(0.98-1.10) | 0.203 | 1.02(0.97-1.08) | 0.447 |
| NHR Q3 | 2340/29601 | 1.09(1.03-1.16) | 0.002 | 1.13(1.07-1.20) | <0.001 | 1.11(1.05-1.18) | <0.001 |
| NHR Q4 | 2182/28820 | 1.08(1.02-1.15) | 0.008 | 1.17(1.11-1.24) | <0.001 | 1.14(1.07-1.21) | <0.001 |
| Pre-DM | 1 |  |  |  |  |  |  |
| NHR Q1 | 224/2487 | 1.21(1.06-1.39) | 0.005 | 1.11(0.97-1.28) | 0.123 | 1.10(0.95-1.28) | 0.197 |
| NHR Q2 | 290/2827 | 1.40(1.24-1.59) | <0.001 | 1.22(1.08-1.37) | 0.002 | 1.22(1.06-1.39) | 0.004 |
| NHR Q3 | 291/2835 | 1.43(1.27-1.62) | <0.001 | 1.19(1.06-1.35) | 0.004 | 1.18(1.03-1.35) | 0.016 |
| NHR Q4 | 302/2947 | 1.50(1.33-1.69) | <0.001 | 1.31(1.16-1.48) | <0.001 | 1.28(1.13-1.46) | <0.001 |
| P for trend |  |  | <0.001 |  | <0.001 |  | <0.001 |

Note: ^*^ Log-transformed NHR; Model 1 adjusted for age, gender, smoker, drinker, education, physical activity. Model 2 adjusted for age, gender, smoker, drinker, education, physical activity, obesity, hypertension, dyslipidemia, C-reactive protein and fasting blood glucose. NHR, neutrophil to high-density lipoprotein cholesterol level ratio; NGR, normal glucose regulation; pre-DM, pre-diabetes mellitus.
